# A novel ruthenium (II)-derived organometallic compound, TQ-6, potently inhibits platelet aggregation: *Ex vivo* and *in vivo* studies

**DOI:** 10.1038/s41598-017-09695-z

**Published:** 2017-08-25

**Authors:** Chih-Hsuan Hsia, Marappan Velusamy, Joen-Rong Sheu, Themmila Khamrang, Thanasekaran Jayakumar, Wan-Jung Lu, Kuan-Hung Lin, Chao-Chien Chang

**Affiliations:** 1 Graduate Institute of Medical Sciences, College of Medicine, Taipei Medical University, Taipei, 110 Taiwan; 2 Department of Chemistry, North Eastern Hill University, Shillong, 793022 India; 3 Department of Medical Research, Taipei Medical University Hospital, Taipei, 110 Taiwan; 4 Central Laboratory, Shin-Kong Wu Ho-Su Memorial Hospital, Taipei, 111 Taiwan; 5 Department of Pharmacology, School of Medicine, College of Medicine, Taipei Medical University, Taipei, 110 Taiwan; 6 Department of Cardiology, Cathay General Hospital, Tai pei, 106 Taiwan

**Keywords:** Biologics, Cardiovascular biology

## Abstract

Arterial thrombosis plays a key role in cardiovascular diseases. Hence, developing more effective antithrombotic agents is necessary. We designed a ruthenium (II)-derived complex, [Ru(η^6^-cymene)2-(1H-benzoimidazol-2-yl)-quinoline Cl]BF_4_ (TQ-6), as a new antiplatelet drug. TQ-6 (0.3 µM) exhibited extremely strong inhibitory activity against platelet aggregation, Src, and Syk phosphorylation stimulated by agonists in human platelets. In collagen-activated platelets, TQ-6 also inhibited ATP-release, [Ca^+2^]i, P-selectin expression, FITC-PAC-1 binding, and hydroxyl radical formation, as well as the phosphorylation of phospholipase Cγ2, protein kinase C, mitogen-activated protein kinases, and Akt. Neither FITC-JAQ1 nor FITC-triflavin binding or integrin β_3_ phosphorylation stimulated by immobilized fibrinogen were diminished by TQ-6. Furthermore, TQ-6 had no effects in cyclic nucleotide formation. Moreover, TQ-6 substantially prolonged the closure time in whole blood, increased the occlusion time of thrombotic platelet plug formation and bleeding time in mice. In conclusion, TQ-6 has a novel role in inhibiting platelet activation through the inhibition of the agonist receptors-mediated inside-out signaling such as Src-Syk-PLCγ2 cascade and subsequent suppression of granule secretion, leading to disturb integrin α_IIb_β_3_-mediated outside-in signaling, and ultimately inhibiting platelet aggregation. Therefore, TQ-6 has potential to develop as a therapeutic agent for preventing or treating thromboembolic disorders.

## Introduction

Platelets are anucleate blood cells that originate from megakaryocytes and play a central role in hemostatic processes. Platelets have an established role in the pathogenesis of atherosclerosis-related diseases including coronary artery diseases and stroke. Under normal conditions, platelets circulate in an inactive form, without significant interactions with the vessel walls. Rupture of an atherosclerotic plaque promotes the activation of platelets and initiates the coagulation cascade^1^. The involvement of platelets in hemostasis is associated with their adherence and aggregation ability; storage granule content release; adsorption, deposition, and transportation of biologically active substances; and endothelial supporting functions^1, 2^. Under high shear conditions in arterial blood vessels, the vascular endothelium is disrupted, and platelets then adhere to the damaged intima and undergo activation. During platelet activation, the release of several mediators [e.g., ADP and thromboxane A_2_ (TxA_2_)] occurs, coupled with intracellular calcium mobilization, thus drawing additional platelets toward the injured endothelium and consequently causing the initial platelet monolayer to thicken. Finally, fibrinogen binds to its specific platelet receptor glycoprotein (GP) IIb/IIIa complex (also known as integrin α_IIb_β_3_), thus completing the final common pathway for platelet aggregation. The complex is formed through calcium-dependent association of GP IIb and GP IIIa, a required step in normal platelet aggregation and endothelial adherence.

Activated platelets are produced TxA_2_, a crucial secondary mediator for further platelet activation. The cyclooxygenase-1 mediated conversion of arachidonic acid (AA) to TxA_2_ can be irreversibly inhibited by aspirin, a standard drug has been used for preventing cardiovascular diseases (CVDs)^3^. Several studies have been focused on searching drugs that target the TxA_2_ pathway in platelets, including various TxA_2_ receptor antagonists (e.g., S-18886)^4^, TxA_2_ synthase inhibitors (e.g., furegrelate)^5^, and compounds act on these two functions (e.g., ridogrel)^6^. Moreover, although aspirin, clopidogrel, and tirofiban are considered as well-established antiplatelet agents on treating thromboembolic diseases, they have still more substantial restrictions when using in clinical settings^7, 8^. Hence, it is important for developing more effective and safe antithrombotic agents to meet the clinical requirements.

**Figure 1:**
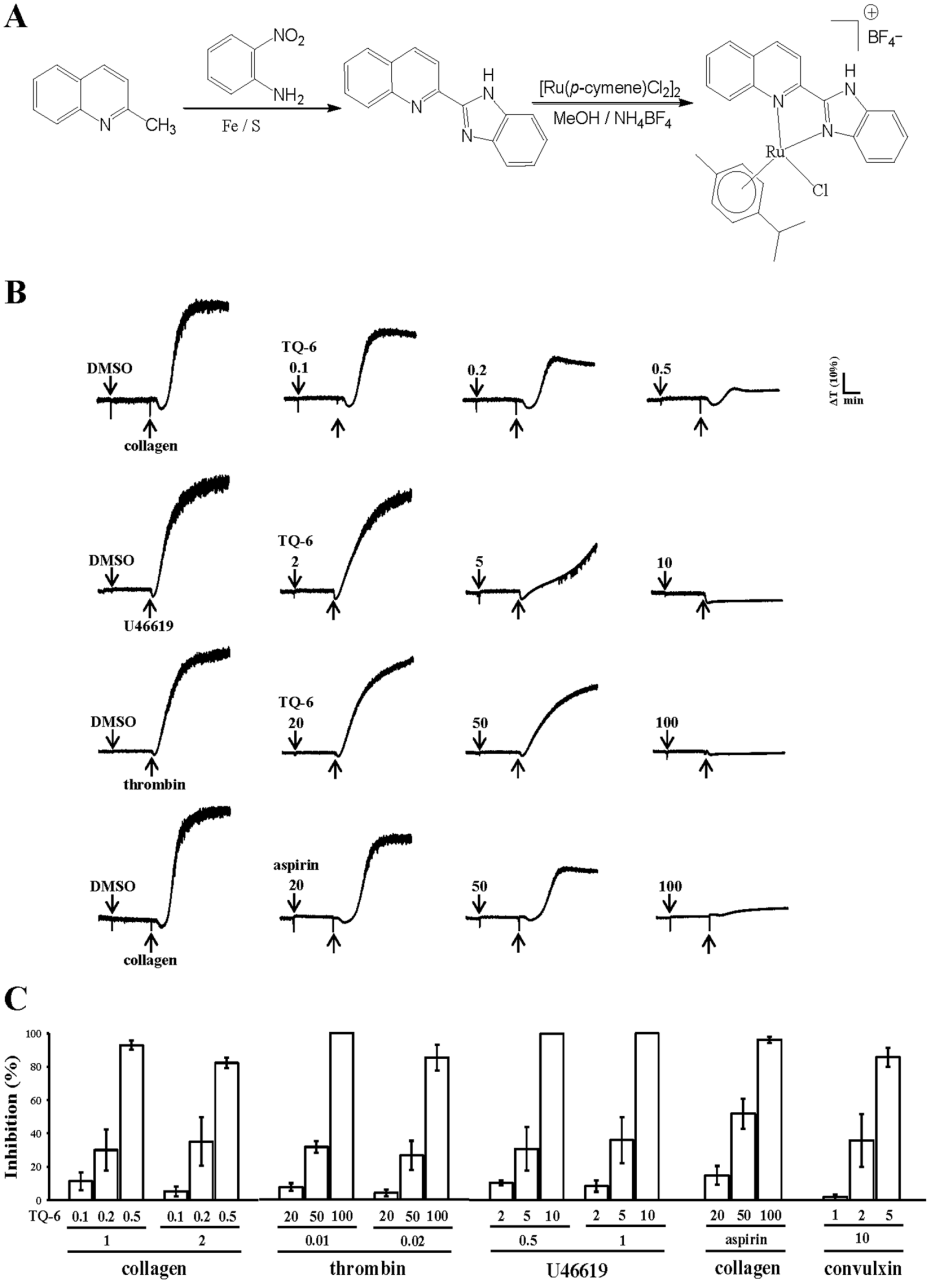
Synthesis of TQ-6 and its inhibitory activity against agonist-induced platelet aggregation in washed human platelets. (**A**) Synthetic process of TQ-6. (**B**) Washed human platelets (3.6 × 10^8^ cells/ml) were preincubated with the solvent control (0.5% DMSO), TQ-6 (0.1–100 μM), or aspirin (20–100 μM) and subsequently treated with 1 μg/ml collagen, 1 μM U46619, and 0.01 U/ml thrombin to stimulate platelet aggregation. **(C)** Concentration-inhibition histograms of TQ-6 and aspirin stimulated by various concentrations of agonists (1 and 2 μg/ml collagen; 0.01 and 0.02 U/ml thrombin; 0.5 and 1 μM U46619; 10 ng/ml convulxin) were presented. Data are presented as means ± standard errors of the means (*n* = 4).

Various metal complexes have been identified over the past decades for anticancer therapy, leading to an increasing amount of related research. Ruthenium derivatives belong to a group of molecules that can be used to synthesize new substances with various biological properties. Currently, researchers have attracted great attention on ruthenium compounds because of their potential antitumor activity with low toxicity toward normal tissues, no cross resistance with cisplatin, and easy absorption by tumor tissue as well as rapid excretion from the body^9–11^. In addition, antiangiogenic therapy is considered a promising strategy for treating cancers. Lai *et al*.^12^ reported that ruthenium complexes have potent antiangiogenic effects through the activation of distinct antiangiogenic signaling pathways. On account of the preceding observations, to discover new biologically active ruthenium derivatives, we developed a new ruthenium compound, TQ-6 (Fig. 1A). Our preliminary findings revealed that TQ-6 exhibits extremely strong inhibitory activity against platelet aggregation *in vitro*. These preliminary results encourage us to further examine the characteristics and functional activity of TQ-6 in platelet activation. This study provides the innovative evidence that the novel ruthenium-derived compound TQ-6 can be developed into a new class of antiplatelet agents.

## Results

### Inhibitory effects of TQ-6 on platelet aggregation in washed human platelets

As shown in Fig. 1B, TQ-6 (0.1, 0.2, and 0.5 μM) strongly and concentration-dependently inhibited platelet aggregation in washed human platelets stimulated by 1 μg/ml collagen. TQ-6 exhibited similar inhibitory activity at 5 and 10 μM against platelet aggregation stimulated by 1 µΜ U46619, a prostaglandin endoperoxide (Fig. 1B). Nevertheless, TQ-6 (50 and 100 μM) showed relatively weak inhibitory activity against 0.01 U/ml thrombin-stimulated platelet aggregation (Fig. 1B). The 50% inhibitory concentration (IC_50_) values of TQ-6 for platelet aggregation induced by collagen, U46619, and thrombin were approximately 0.3, 5, and 60 μM, respectively (Fig. 1C). TQ-6 exhibited more potent activity in inhibiting collagen stimulation than in inhibiting thrombin stimulation. Moreover, aspirin (20, 50, and 100 μM) concentration-dependently inhibited platelet aggregation stimulated by 1 μg/ml collagen, and its IC_50_ value was approximately 50 μM (Fig. 1B and C). The solvent control (0.5% DMSO) did not significantly affect platelet aggregation (Fig. 1B). We have additionally tested two different concentrations of agonists such as collagen, thrombin, and U46619 to verify the impact of TQ-6 on platelet aggregation as shown in Fig. 1C. Besides, convulxin (10 ng/ml), a GP VI agonist^13^, which is purified from the venom of *Crotalusdurissus terrificus* were also examined. As shown in Fig. 1C, there were no noticeable differences found in inhibitory activities of TQ-6 between various concentrations of individual agonists employed (i.e., 1 and 2 µg/ml of collagen, 0.01 and 0.02 U/ml of thrombin, etc). In subsequent experiments, 1 μg/ml collagen was used as an agonist for platelet activation and its related signaling cascades.

### Influence of TQ-6 on cytotoxicity, ATP-release reaction, intracellular calcium mobilization, and surface P-selectin expression

**Figure 2:**
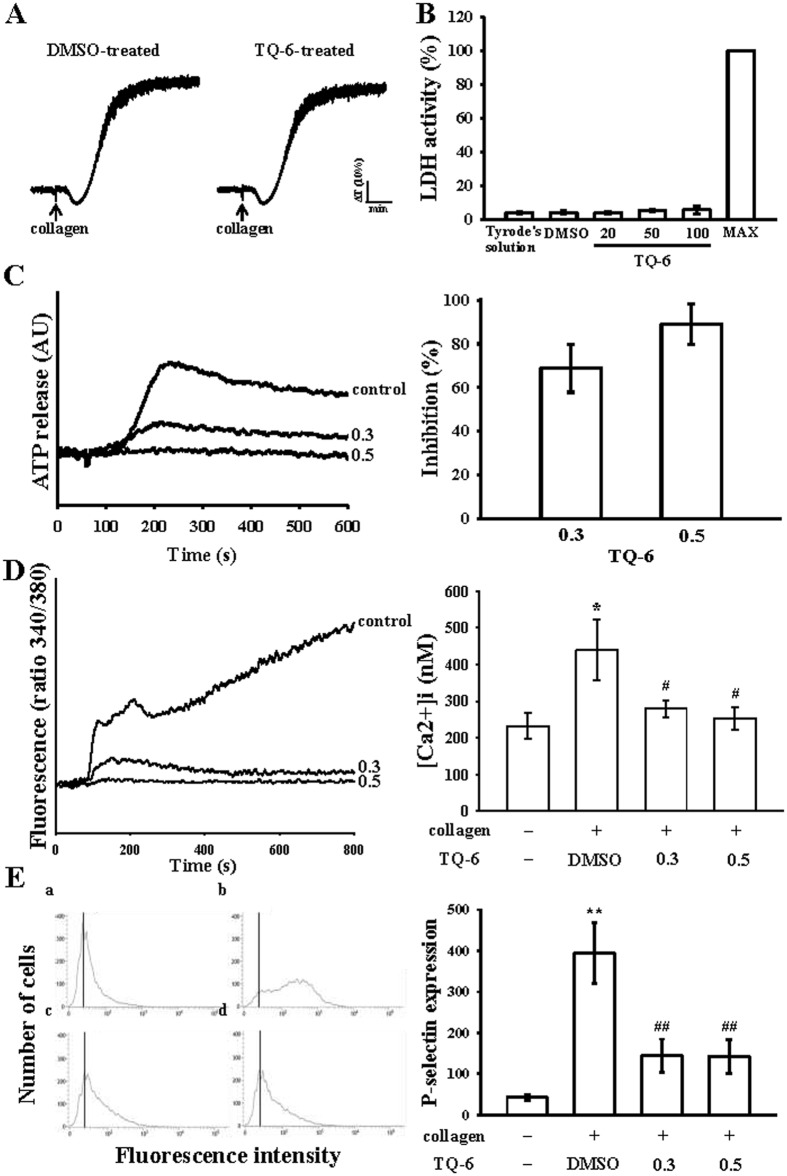
Effects of TQ-6 on cytotoxicity, lactate dehydrogenase (LDH) release, ATP-release reaction, relative [Ca^2+^]i mobilization, and surface FITC-P-selectin expression in human platelets. (**A**) Washed platelets (3.6 × 10^8^ cells/ml) were preincubated with the solvent control (0.5% DMSO) or TQ-6 (100 μM) for 10 min, and subsequently washed two times with Tyrode’s solution, followed by the addition of collagen (1 μg/ml) to trigger platelet aggregation. (**B**) LDH experiment: platelets were preincubated with TQ-6 (20, 50, and 100 µΜ) for 10 min, and a 10-µl aliquot of supernatant was deposited on a Fuji Dri-Chem slide LDH-PIII as described in “Materials and Methods”. Moreover, washed platelets (3.6 × 10^8^ cells/ml) were preincubated with TQ-6 (0.3 and 0.5 µM) or the solvent control (0.5% DMSO), and 1 μg/ml collagen was then added to stimulate either (**C**) ATP-release reaction (AU; arbitrary unit) or (**D**) relative [Ca^2+^]i mobilization (ratio 340/380 nm). (**E**) Washed platelets (3.6 × 10^8^/ml) were preincubated with TQ-6 (0.3 and 0.5 µM) or the solvent control (0.5% DMSO) and FITC-P-selectin (2 µg/ml) for 3 min, and then stimulated by collagen (1 μg/ml). Profiles in (**A**) are representative of four independent experiments. Data in (**B–E**) are presented as means ± standard errors of the means (*n* = 4). *p < 0.05 and **p < 0.01, compared with the resting control; ^#^p < 0.05 and ^##^p < 0.01, compared with the 0.5% DMSO-treated group.

The aggregation curves of platelets that were preincubated with 100 μM TQ-6 for 10 min and subsequently washed two times with Tyrode’s solution demonstrated no significant differences from those of platelets that were preincubated with the solvent control (0.5% DMSO) under equivalent conditions (Fig. 2A), preliminarily indicating that the effects of TQ-6 on platelet aggregation are reversible and noncytotoxic. Furthermore, our LDH study revealed that TQ-6 (20–100 μM) incubated with platelets for 20 min did not significantly increase LDH activity or exert cytotoxic effects on platelets (Fig. 2B), signifying that TQ-6 does not affect platelet permeability or induce platelet cytolysis. Platelet activation is associated with the release of granular contents (e.g., ADP/ATP and calcium from dense granules and surface P-selectin expression from α-granules), thus resulting in ample platelet aggregation. In the present study, TQ-6 (0.3 and 0.5 µM) apparently inhibited both ATP-release reaction (0.3 μM, 68.8 ± 10.9%; 0.5 μM 88.9 ± 9.4%) (Fig. 2C) and relative [Ca^2+^]i mobilization (resting, 232.5 ± 36.3 nM; collagen-activated, 438.9 ± 82.6 nM; 0.3 μM, 279.3 ± 22.6 nM; 0.5 μM, 252.5 ± 31.8 nM; Fig. 2D) in washed human platelets stimulated by 1 μg/ml collagen. The corresponding statistical data were shown in right panels (Fig. 2C and D). In quiescent (resting) platelets, P-selectin is located on the inner wall of α-granules, platelet activation results in the inner walls of the granules are exposed on the outside of the cell^14^. TQ-6 treatment markedly reduced collagen stimulated surface P-selectin expression, the corresponding statistical data was shown in right panel (Fig. 2E),

### Effect of TQ-6 in integrin α_IIb_β_3_ and GP VI activation as well as cyclic nucleotide formation in washed human platelets

**Figure 3:**
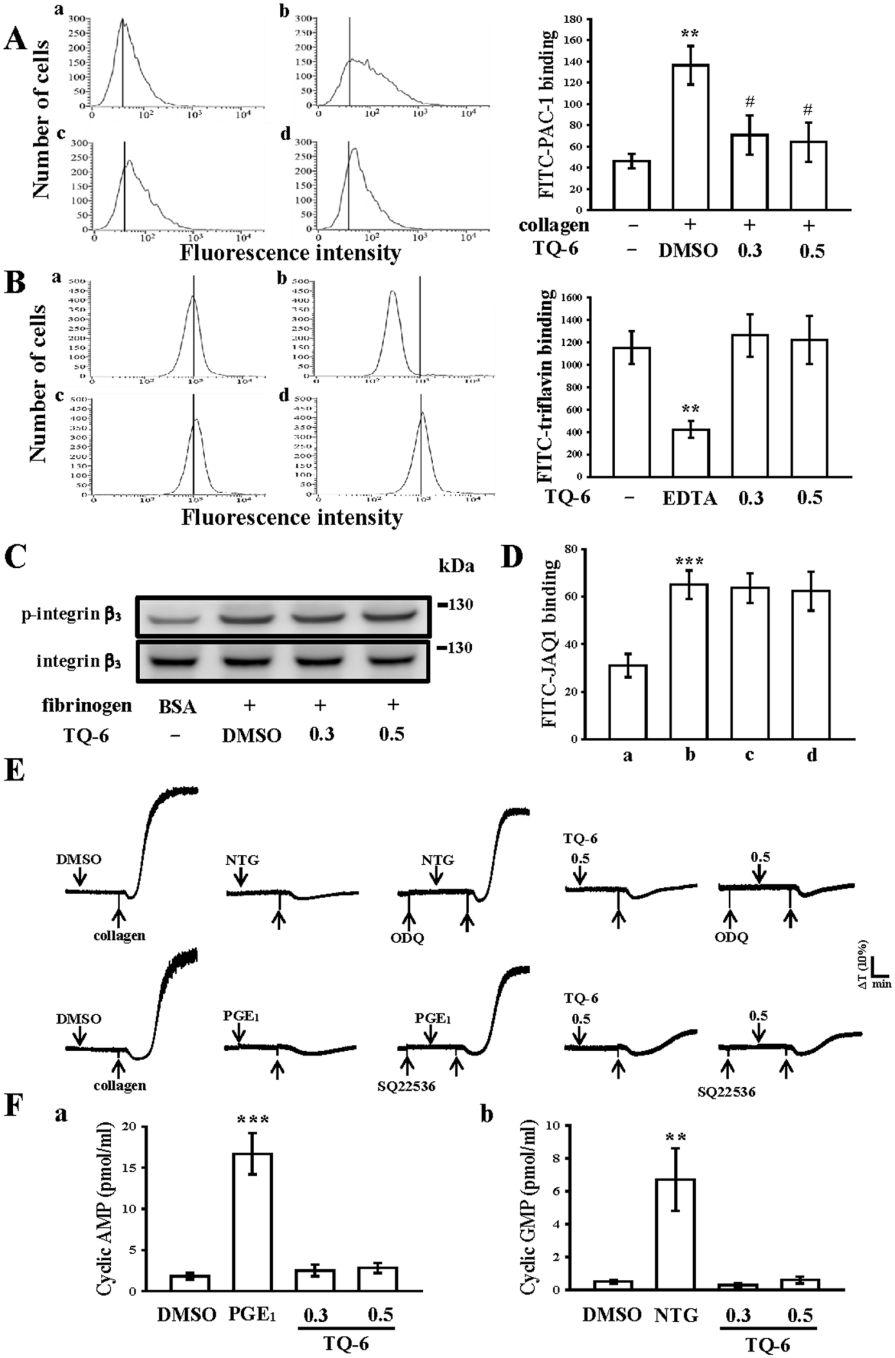
Effects of TQ-6 in integrin α_IIb_β_3_ and GP VI activation as well as cyclic nucleotide formation in human platelets. (**A**) Washed platelets (3.6 × 10^8^/ml) were preincubated with the (b) solvent control (0.5% DMSO) or TQ-6 (c, 0.3 µM; d, 0.5 µM) and FITC-PAC-1 (2 µg/ml) for 3 min in the (a) absence or (b–d) presence of collagen (1 μg/ml). (**B**) Solid line represents the fluorescence profiles of FITC-triflavin (2 µg/ml) serving only as a positive control (a); treatment with EDTA (b, 5 mM; as a negative control), TQ-6 (c, 0.3 µM; d, 0.5 µM), followed by the addition of FITC-triflavin (2 µg/ml). (**C**) Washed platelets were preincubated with the solvent control (0.5% DMSO) or TQ-6 (0.3 and 0.5 µM), and subsequently activated by immobilized fibrinogen (100 μg/ml). Platelets were collected, and their subcellular extracts were analyzed to determine the levels of integrin β_3_ phosphorylation. (**D**) The fluorescence profiles of (a) FITC only as a background control; washed platelets were preincubated with the (b) solvent control (0.5% DMSO) or TQ-6 (c, 0.3 µM; d, 0.5 µM) for 3 min, followed by the addition of FITC-JAQ1 (1 µg/ml). (**E**) For other experiments, washed platelets (3.6 × 10^8^ cells/mL) were preincubated with 10 µM nitroglycerin (NTG), 1 µM prostaglandin E_1_ (PGE_1_), or 0.5 µM TQ-6 with or without 10 µM ODQ or 100 µM SQ22536 and were subsequently treated with 1 µg/ml collagen to induce platelet aggregation. (**F**) Washed platelets were incubated with 0.1 μM PGE_1_, 10 μM NTG, or 0.3 or 0.5 μM TQ-6 for 6 min. The cells were collected, and subcellular extracts were analyzed to determine the levels of cyclic AMP and cyclic GMP, respectively. Profiles in (**C** and **E**) are representative of four independent experiments. Data are presented as means ± standard errors of the means (*n* = 4). **p < 0.01 and ***p < 0.001, compared with the resting control; ^#^p < 0.05, compared with the collagen-treated group.

A large body of evidence suggests that fibrinogen binding to integrin α_IIb_β_3_ is the final step of the common pathway for agonist-induced platelet aggregation. Therefore, we further evaluated whether TQ-6 influences platelet integrin α_IIb_β_3_ activation, leading to the inhibition of platelet aggregation. As shown in the Fig. 3A, TQ-6 significantly affected the binding of FITC-PAC-1 (2 μg/ml) to integrin α_IIb_β_3_ stimulated by collagen (1 μg/ml). The relative fluorescence intensity of binding FITC-PAC-1 in activated platelets was significantly higher than that of the resting platelets (46.1 ± 6.8 vs. 136.3 ± 18.1; Fig. 3A a,b). TQ-6 (0.3 and 0.5 µM) treatment significantly reduced the binding of FITC-PAC-1 to integrin α_IIb_β_3_ (0.3 µM, 70.7 ± 18.6; 0.5 µM, 64.2 ± 18.3; Fig. 3A c,d). Additionally, triflavin, an Arg-Gly-Asp-containing disintegrin purified from *Trimeresurus flavoviridis* venom^15^, inhibits platelet aggregation through direct interference with fibrinogen binding to integrin α_IIb_β_3_^15^. In particular, unlike the binding of fibrinogen or other disintegrins, which requires platelet activation, triflavin binds to resting and activated platelets with similar binding affinities (resting, Kd: 76.0 ± 9.6 nM vs. activated, Kd: 73.5 ± 7.4 nM) and binding numbers^16^. As shown in Fig. 3B, the relative intensity of FITC-triflavin (2 µg/ml) bound to resting platelets was 1154.3 ± 144.3 (*n* = 4), and it significantly decreased in the presence of 5 mM EDTA (negative control, 420.2 ± 75.5, *n* = 4; Fig. 3B a,b). TQ-6 (0.3 and 0.5 µM) had no effects in reduction of FITC-triflavin binding (0.3 µM, 1262.8 ± 189.4; 0.5 µM, 1224.3 ± 214.2, *n* = 4; Fig. 3B c,d) in resting platelets. For better elucidation of the mechanisms by whether TQ-6 impaired integrin α_IIb_β_3_-mediated outside-in signaling, the integrin β_3_ phosphorylation, a vital indicator of outside-in signaling, was chosen for further experiments. We examined integrin β_3_ phosphorylation in platelets exposed to immobilized fibrinogen by immunoblotting assay, and found that phosphorylation of integrin β_3_ was not attenuated by TQ-6 (0.3 and 0.5 μM) (Fig. 3C). Taken together, these results indicate that inhibitory mechanisms of TQ-6 in platelet aggregation may not directly act in integrin α_IIb_β_3_-mediated outside-in signaling, but it may be regulated by other inside-out signaling to disturb the integrin α_IIb_β_3_ activation.

GP VI is the major collagen receptor that mediates platelet adhesion and aggregation^17^. We further evaluated whether TQ-6 inhibits platelet activation by specifically binding to GP VI on the platelet membrane. As shown in Fig. 3D, the relative fluorescence intensity of FITC-JAQ1 (mAb raised against GP VI; 1 μg/ml) bound directly to the platelets was obviously higher than that of the background control (FITC only). FITC-JAQ1 binding was not diminished in the presence of TQ-6 (0.3 and 0.5 µM) (Fig. 3D), and JAQ1 may target the collagen binding site in the GP VI molecule, indicating that TQ-6 did not directly act on GP VI in human platelets.

In addition, both 10 μM ODQ, a guanylate cyclase inhibitor, and 100 μM SQ22536, an adenylate cyclase inhibitor, significantly reversed the inhibition of collagen-induced platelet aggregation mediated by 10 μM nitroglycerin (NTG) and 1 μM PGE_1_ (Fig. 3E). Neither ODQ nor SQ22536 significantly reversed the inhibition of collagen-induced platelet aggregation mediated by 0.5 μM TQ-6 (Fig. 3E). As shown in the Fig. 3F, levels of cyclic AMP and cyclic GMP in resting platelets were lower compared with those of 1 μM prostaglandin E_1_ (PGE_1_)- and 10 μM NTG-treated platelets, respectively (1.8 ± 0.4 vs. 16.7 ± 2.5 pmol/ml, *n* = 4, Fig. 3F a; 0.5 ± 0.1 vs. 6.7 ± 1.9 pmol/ml, *n* = 4, Fig. 3F b). Neither cyclic AMP nor cyclic GMP level was significantly increased after TQ-6 treatment (0.3 and 0.5 μM) (Fig. 3F a,b). These results indicated that the mechanism of TQ-6-mediated inhibition of platelet aggregation appears to be independent of the increasing formation of cyclic nucleotides [e.g., cyclic AMP (cAMP) and cyclic GMP (cGMP)].

### Effects of TQ-6 on Src, Syk, and phospholipase Cγ2/protein kinase C cascade activation

**Figure 4:**
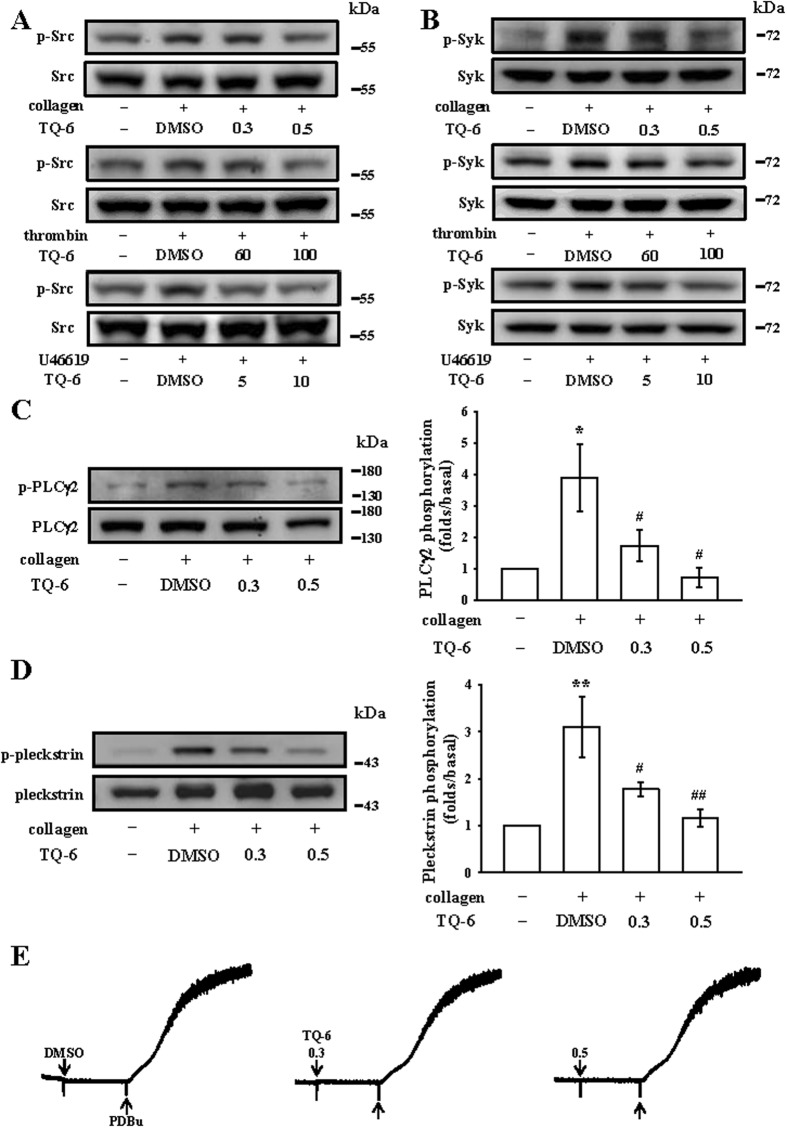
Regulatory effects of TQ-6 on the Src, Syk, and PLCγ2-PKC activation in platelets. Washed platelets were preincubated with TQ-6 or the solvent control (0.5% DMSO) and subsequently treated with either 1 μg/ml collagen, 0.01 U/ml thrombin, 1 μM U46619 or 150 nM PDBu to induce (**A**) Src, (**B**) Syk, (**C**) PLCγ2, (**D**) PKC activation (pleckstrin phosphorylation) or (**E**) platelet aggregation. Profiles in (**A,B**, **E**) are representative of four independent experiments. Data are presented as means ± standard errors of the means (*n* = 4). *p < 0.05 and **p < 0.01, compared with the 0.5% DMSO-treated group; ^#^p < 0.05 and ^##^p < 0.01, compared with the collagen-treated group.

Src family kinases (SFK; such as Src, Fyn) play a central role in mediating the rapid response of platelet activation. Ligand-mediated of diverse receptors triggers an increase in SFK activity and downstream tyrosine phosphorylation of enzymes, adaptors, and cytoskeletal proteins that collectively propagate the signal and coordinate platelet activation^18^. The Src family kinases are activated through phosphorylation of tyrosine based activation and subsequently leading to binding and activation of the tyrosine kinase Syk. TQ-6 manifestly reduced both SFK (i.e., Src) and Syk phosphorylation, respectively, in washed platelets stimulated by collagen, thrombin, and U46619 (Fig. 4A and B). Additionally, phospholipases C (PLCs) hydrolyze phosphatidylinositol 4,5-bisphosphate to generate the secondary messengers inositol 1,4,5-trisphosphate (IP_3_) and diacylglycerol (DAG). IP_3_ triggers intracellular Ca^2+^ mobilization and DAG activates protein kinase C (PKC) activation, inducing a protein with an apparent molecular weight of approximately 47 kDa, which is predominantly phosphorylated (p47 protein; pleckstrin) and causes the ATP-release reaction^19^. As presented in the Fig. 2C and D, we demonstrated the inhibitory effects of TQ-6 against ATP release and intracellular Ca^2+^ mobilization. We further investigated the influence of TQ-6 on the phosphorylation of the PLCγ2-PKC signaling cascade. At 0.3 and 0.5 µM, TQ-6 evidently reduced both PLCγ2 phosphorylation and PKC activation (pleckstrin phosphorylation), respectively, in collagen-activated platelets (Fig. 4C and D). In addition, neither 0.3 nor 0.5 µM TQ-6 significantly affected platelet aggregation induced by 150 nM phorbol 12,13-dibutyrate (PDBu), a PKC activator (Fig. 4E), signifying that although TQ-6 does not directly affect PKC activation, it may interfere with the upstream regulator of PLCγ2.

### Inhibitory effects of TQ-6 on MAPK and Akt phosphorylation

**Figure 5:**
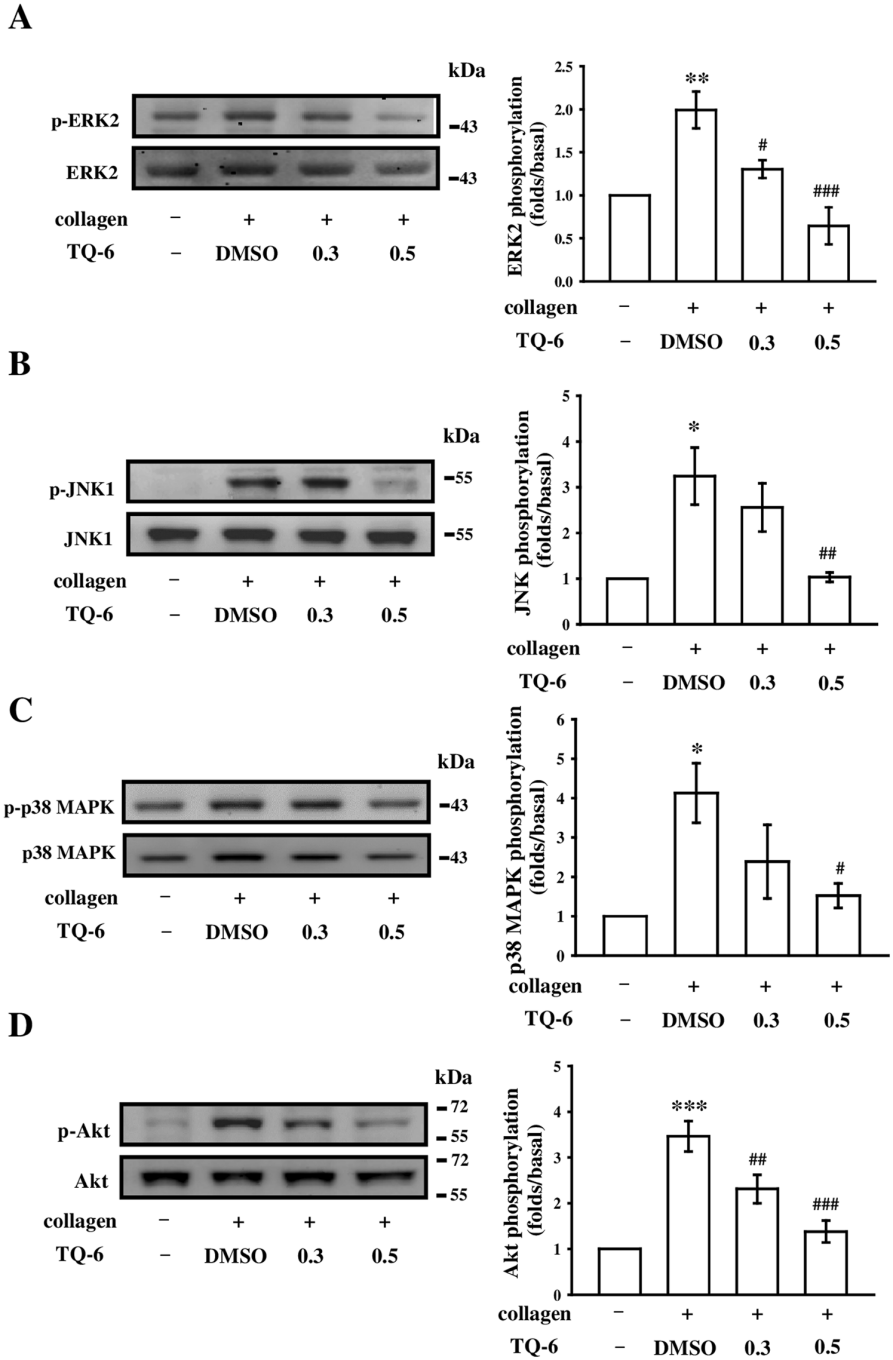
Effects of TQ-6 on ERK2, JNK1, p38 MAPK, and Akt phosphorylation in collagen-activated platelets. Washed platelets (1.2 × 10^9^ cells/ml) were preincubated with 0.3 or 0.5 μM TQ-6 or the solvent control (0.5% DMSO) and subsequently treated with 1 μg/ml collagen to induce platelet activation. Platelets were collected, and their subcellular extracts were analyzed to determine the levels of (**A**) ERK2, (**B**) JNK1, (**C**) p38 MAPK, and (**D**) Akt phosphorylation. Data are presented as means ± standard errors of the means (*n* = 4). *p < 0.05, **p < 0.01, and ***p < 0.001, compared with the solvent control (0.5% DMSO) group; ^#^p < 0.05, ^##^p < 0.01, and ^###^p < 0.001, compared with the collagen-treated group.

To investigate the inhibitory mechanisms of TQ-6 in platelet activation, we detected several signaling molecules of the MAPK and Akt phosphorylation pathways. MAPKs, including extracellular signal-regulated kinases 1 and 2 (ERK1/2), c-Jun N-terminal kinases 1 and 2 (JNK1/2), and p38 MAPK, control major cellular responses in eukaryotic organisms and contribute to cell proliferation, migration, differentiation, and apoptosis. ERK2, JNK1, and p38 MAPK have been identified in platelets^20^. Notably, 0.5 μM TQ-6 significantly inhibited the phosphorylation of ERK2, JNK1, and p38 MAPK (Fig. 5). Moreover, Akt, also known as protein kinase B, is a serine/threonine-specific protein kinase that plays a key role in multiple cellular processes such as platelet activation, cell proliferation, apoptosis, and cell migration^21^. In this study, TQ-6 concentration-dependently inhibited collagen-induced Akt phosphorylation (Fig. 5D), indicating that the inhibition of the MAPKs and Akt signaling pathways is crucial in the TQ-6-mediated inhibition of platelet activation.

### Regulatory roles of TQ-6 in hydroxyl radical formation by ESR spectrometry and thrombus formation ***in vivo***

**Figure 6:**
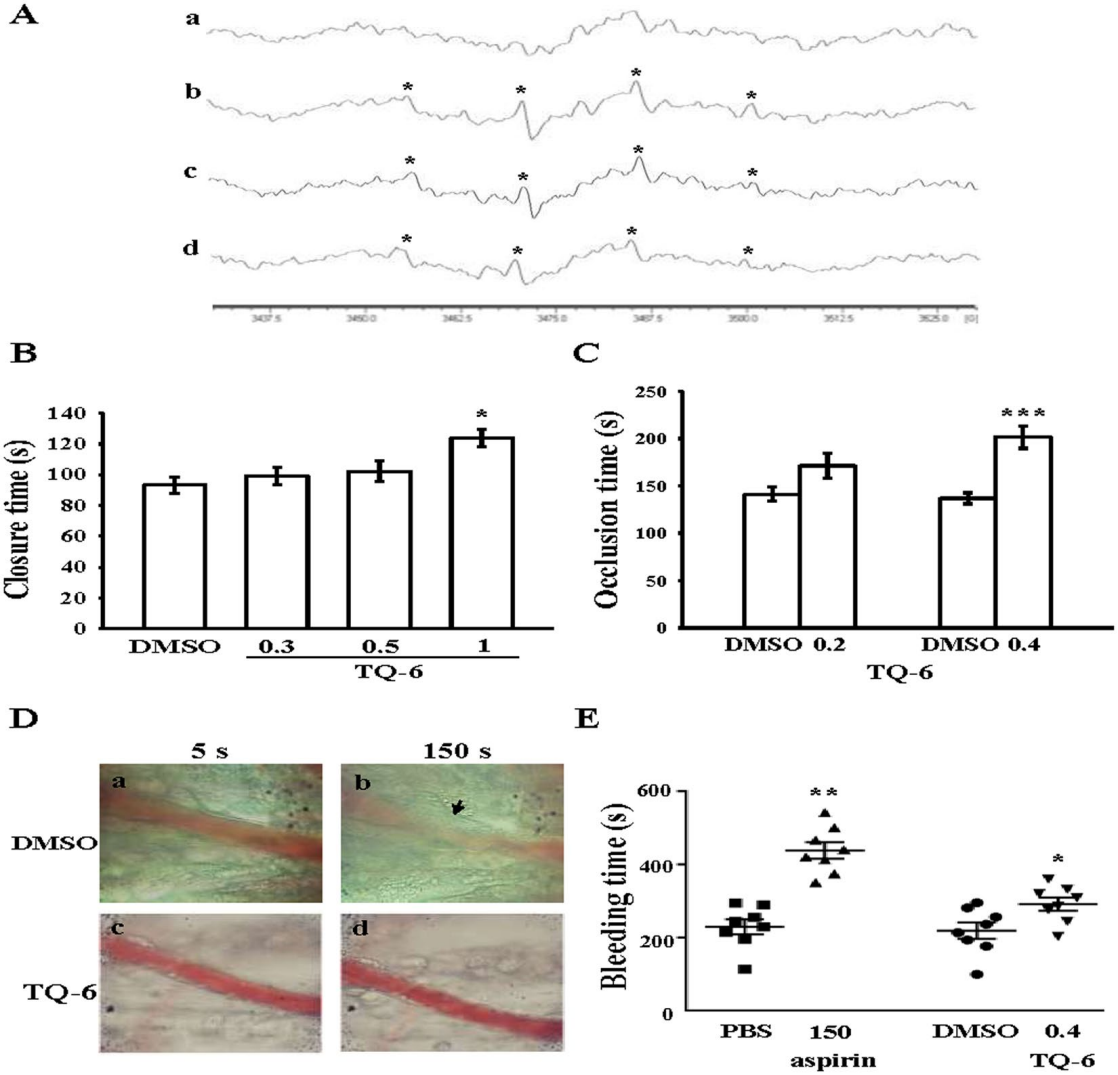
Protective effects of TQ-6 on OH**·** formation by electron spin resonance (ESR) study, closure time according to the analysis on the PFA-100 system and thrombotic platelet plug formation in the mesenteric venules of mice as well as the bleeding time in mouse tail vein. (**A**) Washed platelets were preincubated with Tyrode’s solution (a, resting control) or treated with (b) 0.5% DMSO or TQ-6 at (c) 0.3 µM or (d) 0.5 µM, followed by the addition of collagen (1 µg/ml) to trigger OH**·** formation. Profiles are representative of four independent experiments and an asterisk (*) indicates the formation of OH·. (**B**) Shear-induced platelet plug formation in whole blood was determined by recording the closure time of CEPI-coated membranes, as described in the “Materials and methods.” (**C**) For another study, mice were administered an intravenous bolus of the solvent control (0.5% DMSO) or TQ-6 (0.4 mg/kg), and the mesenteric venules were irradiated to induce microthrombus formation (occlusion time). (**D**) Microscopic images (400 × magnification) of 0.5% DMSO-treated controls (a, b) and the TQ-6 (0.4 mg/kg)-treated groups (c, d) were recorded at 5 (a, c) and 150 s (b, d) after irradiation. Photographs are representative of six similar experiments. Arrow indicates platelet plug formation. (**E**) The bleeding time was measured through transection of the tail in mice after 30 min of administering either 0.4 mg/kg TQ-6 or 150 mg/kg aspirin intraperitoneally. Data are presented as means ± standard errors of the means (B,C, *n* = 6; E, *n* = 8). *p < 0.05, **p < 0.01, and ***p < 0.001, compared with the solvent control (0.5% DMSO or PBS)-treated group.

An ESR signal indicative of hydroxyl radical (OH**·**) formation was observed in collagen-activated platelets, but not in resting platelets (Fig. 6A a,b). In this study, we demonstrated that incubation of human platelets with collagen produced a typical four-line hydroxyl radical (OH·) signal (a^N^ = a^H^ = 14.8 G) and long lived g = 2.005 radical detectable by the spin trapped DMPO (Fig. 6A b), but this four-line signal was not detected in resting platelets (Fig. 6A a). Treatment with 0.3 and 0.5 μM TQ-6 considerably diminished the four-line OH**·**ESR signal by approximately 20% and 41%, respectively, compared with the collagen-treated platelets (Fig. 6A c,d).

Shear-induced platelet plug formation in whole blood was analyzed *ex vivo*. The PFA-100 system was used to mimic the *in vivo* conditions of blood vessel injury, in which platelets were exposed to a high shear rate to record the time required for platelet aggregation to occlude an aperture in a collagen-coated membrane. The CT of the CEPI-coated membrane in whole blood treated with the solvent control (0.5% DMSO) was 93.2 ± 5.5 s (*n* = 6) (Fig. 6B). Treatment with 1 μM TQ-6 significantly increased the CT of the CEPI-coated membranes (123.8 ± 5.7 s, *n* = 6, p < 0.05; Fig. 6B), indicating that the adherence of platelets to collagen prolonged under flow conditions after 1 μM TQ-6 treatment. Furthermore, we directly investigated the inhibitory effect of TQ-6 on thrombus formation *in vivo*. The occlusion time in the mesenteric microvessels of mice pretreated with 15 µg/kg fluorescein sodium was approximately 180 s. When TQ-6 was administered at 0.2 or 0.4 mg/kg after pretreatment with fluorescein sodium, the occlusion times were significantly prolonged after 0.4 mg/kg TQ-6 treatment as compared with those of DMSO-treated controls (ctl, 141.1 ± 7.5 s vs. 0.2 mg/kg, 171.1 ± 13.1 s, *n* = 6, p > 0.05; ctl, 136.6 ± 5.7 s vs. 0.4 mg/kg, 201.5 ± 12.0 s, *n* = 6, p < 0.001) (Fig. 6C). After irradiation, a thrombotic platelet plug was observed in mesenteric microvessels at 150 s, but not at 5 s, in the DMSO-treated group (Fig. 6D a,b). On administering 0.4 mg/kg TQ-6, platelet plug formation was not observed at 5 or 150 s after irradiation (Fig. 6D c,d). The observed blood flow rate in the control venule was lower than that in the TQ-6-treated venule, because the platelet plug appeared in the control venule at 150 s (Fig. 6D b).

In the tail transection model of mice, after 30 min of administering 150 mg/kg aspirin intraperitoneally, the bleeding time considerably increased from 229.2 ± 20.5 s (PBS-treated control group) to 438.1 ± 22.6 s. The bleeding time of the 0.4 mg/kg TQ-6-treated mice was slightly longer than that of the solvent control (0.5% DMSO)-treated mice (218.7 ± 22.5 s vs. 291.2 ± 17.8 s) (Fig. 6E). In this study, each animal was monitored for 10 min even if bleeding stopped, in order to detect any re-bleeding.

## Discussion

Ruthenium complexes are an attractive alternative for platinum because they have several favorable properties suitable for rational anticancer drug design and biological applications. Thus, ruthenium metal complexes are considered one of the most promising anticancer agents^22^. Notably, our results show, for the first time, that in addition to its antitumor activity, TQ-6, a novel synthetic ruthenium (II)-derived compound, exhibits highly potent antiplatelet activity (IC_50_, 0.3 µM) *ex vivo* and effectively inhibits arterial thrombogenesis (0.4 mg/kg) *in vivo*. Platelets are activated by various physiological stimuli (e.g., collagen and thrombin). These agonists are considered to exert their effects by interacting with specific receptors on platelet membranes. According to the IC_50_ values, TQ-6 had the stronger inhibitory activity against collagen-stimulated platelet aggregation than did U46619; it also presented relatively weak activity against thrombin stimulation, indicating that TQ-6 inhibits platelet aggregation through a considerable SFK-Syk-PLCγ2/PKC-dependent mechanism. Aspirin has been clinically used for treating and preventing CVDs. Our evaluation demonstrated that under identical conditions, TQ-6 was more potent than aspirin, indicating its potential clinical applications.

Platelets adhere to subendothelial matrix proteins (i.e., collagen), thus changing their shape and causing the release of granular contents (e.g., ATP, Ca^+2^, and P-selectin). Collagen, present in the vascular subendothelium and vessel wall, acts as a platelet adhesion substrate as well as an endogenous platelet activator. Among the platelet receptors interacting directly with collagen, integrin α_2_β_1_ (GP Ia/IIa) and GP VI might contribute to the overall processes of platelet adhesion and activation^23^. Signals (e.g., tyrosine phosphorylation and matrix remodeling) are activated in platelets expressing integrin α_2_β_1_ after cell adhesion to collagen^24^. GP VI ligands, including collagen and convulxin, induce receptor clustering, which facilitates the phosphorylation of the tandem tyrosine found in the immunoreceptor tyrosine-based activation motifs of the noncovalently associated Fcγ-chain receptor adaptor by SFKs (e.g., Src and Lyn)^25^. TQ-6 inhibited platelet aggregation at different degrees, depending on the agonists (collagen, U46619, and thrombin), thus indicating that TQ-6 was potentially not effective on specific/individual receptors of these agonists (i.e., GP VI). Thus, TQ-6 probably acts through a common signal cascade against stimulated platelets. A major component of the platelet aggregation response is the binding of fibrinogen to integrin α_IIb_β_3_ on activated platelets. Triflavin acts as a specific antagonist for integrin α_IIb_β_3_, which interferes with the fibrinogen–integrin α_IIb_β_3_ interaction^15^. In this study, TQ-6 significantly affected the binding efficiency of FITC-PAC-1 to activated integrin α_IIb_β_3_ without influencing upon the FITC-triflavin binding. Besides, integrin β_3_ phosphorylation stimulated by immobilized fibrinogen was not alleviated by TQ-6, which infers that the antiplatelet activity of TQ-6 is not directly interference the binding of fibrinogen toward to integrin α_IIb_β_3_ and its mediated outside-in signaling. Furthermore, TQ-6 inhibited ATP release and [Ca^+2^]i mobilization from intracellular granules, corroborating the concept that changes in ATP and cytosolic Ca^+2^ play a major role in platelet activation.

Human platelet activation is inhibited through intracellular cAMP- and cGMP-mediated pathways, and the importance of cyclic nucleotides in modulating platelet activation is firmly established^26^. At elevated levels, cyclic nucleotides inhibit most platelet responses and reduce [Ca^2+^]i levels through Ca^2+^ uptake by the dense tubular system, thereby suppressing PLC and PKC activation^26^. Thus, cAMP and cGMP act synergistically to inhibit platelet activation. We observed that neither SQ22536 nor ODQ significantly reversed the TQ-6-mediated inhibition of collagen-induced platelet aggregation and even at a maximal concentration of 0.5 µM TQ-6 did not affect the increasing intracellular levels of cAMP or cGMP in human platelets (Fig. 3F). Therefore, TQ-6-mediated inhibition of platelet activation is independent of intracellular cyclic nucleotide formation.

Ligand-mediated agonist receptors triggers transmission of primary activation signals through the phosphorylation of downstream tyrosine residues in proteins. None of these receptors have intrinsic kinase activity; instead, they rely on SFK (e.g., Src, Lyn, Fyn) activation that are either associated with or in close proximity to their cytoplasmic tails, to transmit signals. Downstream effectors of SFK include adaptors, enzymes (i.e., Syk), and cytoskeletal proteins that collectively coordinate cytoskeletal remodeling, degranulation, and integrin activation, which is referred to as inside-out signaling^18^. Moreover, platelet activation by agonists, such as collagen, substantially alters phospholipase activation. PLC activation results in IP_3_ and DAG production, which activates PKC, thus inducing p47 phosphorylation^19^. PKC activation enables particular responses that facilitate the transmission of specific activating signals in distinct cellular compartments. PLCs can be classified into six families: PLCβ, PLCγ, PLCδ, PLCε, PLCζ, and PLCη^27^. The PLCγ family comprises the isozymes PLCγ1 and PLCγ2. PLCγ2 is involved in collagen-dependent signaling in platelets^28^. TQ-6 evidently diminished collagen-induced PLCγ2-PKC activation; however, TQ-6 had no direct effects on PKC activation because it did not inhibit PDBu-induced platelet aggregation, suggesting that TQ-6-mediated inhibition of platelet activation involves PLCγ2 downstream signaling. This result also explains how TQ-6 is more efficacious in inhibiting collagen-induced platelet aggregation than that induced by thrombin.

Akt is a downstream effector of phosphoinositide 3-kinase (PI3K); Akt-knockout mice have been reported to exhibit defects in agonist-induced platelet activation, suggesting that Akt regulates platelet activation, with potential consequences for thrombosis^21, 29^. Therefore, selective inhibitors of Akt isoforms or of proteins contributing to its activation, such as individual PI3K isoforms, may be attractive targets for antithrombotic therapy^21^. In addition, MAPKs are activated by specific MAPK kinases (MEKs); specifically, MEK1/2 activates ERK1/2, MEK3/6 activates p38 MAPK, and MEK4/7 activates JNK1/2^30^. ERK2 activation was reported to be involved in platelet aggregation induced by low-dose collagen, requiring prior ATP release, which triggers P_2_ X_1_-mediated Ca^2+^ influx and activates ERK2, thereby increasing myosin light-chain kinase phosphorylation^31^. Cytosolic phospholipase A_2_ (cPLA_2_) is a substrate of p38 MAPK activity induced by various agonists such as von Willebrand factor (VWF), thrombin, and collagen^31^. Thus, p38 MAPK is essential for the stimulation of cPLA_2_ and release of AA, leading to TxA_2_ production in platelets^32^. JNK1 is the most recently identified MAPK in platelets, and its activation or role is therefore poorly defined. JNK1 is activated by several agonists such as thrombin, VWF, collagen, and ADP^31^, and JNK1 activation was reported to trigger integrin α_IIb_β_3_ activation^31^. In the current study, ERK2, JNK1, and p38 MAPK, appear to play a crucial role in TQ-6-mediated inhibition of platelet activation.

Reactive oxygen species derived from platelet activation (i.e., hydrogen peroxide and OH**·**) might affect cells they come in contact with, such as endothelial cells; this can amplify platelet reactivity during thrombus formation. Free radical species act as secondary signals that increase [Ca^2+^]i levels during the initial phase of platelet activation, and PKC is involved in receptor-mediated free radical production in platelets^33^. In addition, hydrogen peroxide produced by platelets is converted into OH**·** because platelet aggregation can be inhibited by OH**·** scavengers^33^. Our ESR spectrometry results provide direct evidence that TQ-6 diminished OH**·** formed through collagen stimulation in platelets. Thus, the *in vivo* TQ-6-mediated inhibition of thrombogenesis may involve free radical scavenging. After vascular endothelial cell injury, exposure to subendothelial collagen is the major trigger initiating platelet adhesion and aggregation at the site of injury, followed by arterial thrombus formation. The PFA-100 system records the time required for platelet aggregation to occlude an aperture in a collagen-coated membrane. Platelet adhesion to collagen depends on flow conditions; in this study, platelets prolonged adhesion to collagen under flow conditions. In a thrombosis study, the mesenteric venules were continuously irradiated by fluorescein sodium throughout the experimental period, leading to strong damage to the endothelial cells^34^. TQ-6 significantly prolonged both closure times and occlusion times; these effects may be mediated, at least partly, by the inhibition of platelet activation. In addition, the tail transection model of mice was used to examine the effect of TQ-6 on bleeding time *in vivo*. Although aspirin is the most effective antiplatelet drug prescribed for preventing or treating cardiovascular and cerebrovascular diseases, it causes an unwanted prolongation of bleeding time. In the tail transection model, the slight prolongation of the bleeding time may be caused by the antiplatelet activity of TQ-6, at least partly. Furthermore, these results may serve basically as a general example of how TQ-6 increases bleeding time compares to prominent bleeding phenotypes.

In conclusion, the findings of this study reveal that TQ-6 has a novel role in inhibiting platelet activation through the inhibition of the agonist receptors-mediated inside-out signaling such as SFK-Syk-PLCγ2-PKC cascade and subsequent suppression of Akt and MAPKs activation. These alterations reduce granule secretion (i.e., ATP release, [Ca^2+^]i levels, and surface P-selectin expression), leading to disturb integrin α_IIb_β_3_-mediated outside-in signaling (i.e., integrin β_3_ phosphorylation), and ultimately inhibiting platelet aggregation. However, we could not rule out the involvement of other mechanisms, which are yet to be identified, in TQ-6-mediated inhibition of platelet activation. Thus, our findings suggest a novel ruthenium derivative, TQ-6, as a potential therapeutic agent for preventing or treating thromboembolic disorders.

## Materials and Methods

### Chemicals and Reagents

Collagen, luciferin-luciferase, U46619, phorbol 12,13-dibutyrate (PDBu), heparin, prostaglandin E_1_ (PGE_1_), aspirin, 5,5-dimethyl-1-pyrroline N-oxide (DMPO), SQ22536, 1H-[1,2,4]oxadiazolo[4,3-a]quinoxalin-1-one (ODQ), bovine serum albumin (BSA), fibrinogen, and thrombin were purchased from Sigma (St. Louis, MO, USA). Fura-2AM, an intracellular calcium indicator, was purchased from Molecular Probes (Eugene, OR, USA). Convulxin, anti-phospho-p38 mitogen-activated protein kinase (MAPK) Ser^182^, anti-integrin β_3_ monoclonal antibodies (mAbs), and anti-phospho-integrin β_3_ (Tyr^759^) polyclonal antibody (pAb) were purchased from Santa Cruz Biotechnology (Santa Cruz, CA, USA). Anti-p38 MAPK, anti-phospho-JNK (Thr^183^/Tyr^185^), anti-Syk (D3Z1E) XP®, anti-phospho-Src family (Tyr^416^), and anti-p44/42 ERK (1/2) mAbs, as well as anti-PLCγ2, anti-phospho (Tyr^759^) PLCγ2, anti-phospho-(Ser) PKC substrate (pleckstrin; p-p47), anti-JNK, anti-phospho-Syk (Tyr^525/526^), anti-Src family, and anti-phospho-p44/p42 ERK (Thr^202^/Tyr^204^) pAbs, were purchased from Cell Signaling (Beverly, MA, USA). Anti-phospho-Akt (Ser^473^) and anti-Akt mAbs were purchased from Biovision (Mountain View, CA, USA). An anti-pleckstrin (p47) pAb was purchased from GeneTex (Irvine, CA, USA). A Hybond-P PVDF membrane, an enhanced chemiluminescence Western blotting detection reagent, horseradish peroxidase (HRP)-conjugated donkey anti-rabbit immunoglobulin G (IgG), and sheep anti-mouse IgG were purchased from Amersham (Buckinghamshire, UK). Fluorescein isothiocyanate (FITC)-labelled anti-GP VI (JAQ1) mAb was obtained from Emfret Analytics (Würzburg, Germany). FITC anti-human CD42P (P-selectin) and FITC anti-human CD41/CD61 (PAC-1) mAbs were obtained from BioLegend (San Diego, CA, USA). Cyclic AMP and cyclic GMP enzyme immunoassay (EIA) kits were obtained from Cayman (Ann Arbor, MI). The Dade Behring PFA-100 collagen/epinephrine (CEPI) test cartridge was obtained from Siemens Healthcare (Erlangen, Germany).

### TQ-6 synthesis. 2-(1H-Benzoimidazol-2-yl)-quinoline (L)

2-Methyl-quinoline (2.86 g, 20 mM) was added to a Schlenk flask containing 2-nitro-phenylamine (1.38 g, 10 mM), elemental sulfur (32 mg, 1 mM), and iron powder (56 mg, 1 mM). After the flask was flushed with argon, the contents were stirred at 160 °C under an atmosphere of argon for 24 h. The reaction mixture was then allowed to cool down, excess iron powder was removed by filtration through a celite pad, and the solvent was evaporated under reduced pressure. Next, the residue was dissolved in dichloromethane, washed with water, dried over anhydrous Na_2_SO_4_, and concentrated through rotary evaporation. The resulting crude product was subjected to silica gel column chromatography and eluted with DCM/MeOH (99:1) to afford the pure title compound L: pale yellow solid (1.6 g, 65%); mp 218 °C–220 °C; ^1^H NMR (400 MHz, CDCl_3_) δ 10.97 (s, 1 H), 8.50–8.48 (d, 1 H, *J* = 8 Hz), 8.26–8.24 (d, 1 H, *J* = 8 Hz), 8.05–8.02 (d, 1 H, *J* = 12 Hz), 7.83–7.79 (t, 2 H, *J* = 8 Hz), 7.71–7.66 (m, 1 H), 7.53–7.49 (t, 1 H, *J* = 8 Hz), 7.43–7.41 (d, 1 H, *J* = 8 Hz), 7.25–7.21 (m, 2 H); ESI-MS m/z 244 (M^+^).

### [Ru(η^6^-cymene) 2-(1H-benzoimidazol-2-yl)-quinoline Cl] BF_4_ (TQ-6)

[Ru(p-cymene)(Cl)_2_]_2_ (0.12 g, 0.2 mM) and **L** (0.1 g, 0.4 mM) were suspended in methanol (20 ml) and stirred at room temperature for 2 h. NH_4_BF_4_ (200 mg, 0.6 mM) in MeOH (10 ml) was added to the initially yellow solution, thus changing its color to orange. After 24 h, the solution was evaporated, and the resulting solid was filtered out. The residue was washed with diethyl ether (40 ml) and dried under vacuum. The desired products were recrystallized from DCM:hexane solvent mixture to give orange microcrystals: ^1^H NMR (400 MHz, DMSO-D_6_) δ 15.46 (br s, 1 H), 8.98–8.96 (d, 1 H, *J* = 8 Hz), 8.83–8.80 (d, 1 H, *J* = 12 Hz), 8.66–8.64 (d, 1 H, *J* = 8 Hz), 8.29–8.27 (d, 1 H, *J* = 8 Hz), 8.19–8.13 (m, 2 H), 7.97–7.94 (t, 2 H, *J* = 6 Hz), 7.90–7.87 (m, 2 H), 6.33–6.32 (d, 2 H, *J* = 4 Hz), 6.31–6.29 (d, 1 H, *J* = 8 Hz), 6.11–6.09 (d, 1 H, *J* = 8 Hz), 2.28 (s, 3 H), 2.21–2.15 (m, 1 H), 0.73 (s, 6 H); ESI-MS: *m/z* = 516 [M-BF_4_]^+^ (Fig. 1A). The freshly obtained TQ-6 was dissolved in DMSO and stored at 4 °C.

### Platelet aggregation

This study was approved by the Institutional Review Board of Taipei Medical University (TMU-JIRB-N201612050) and conformed to the directives of the Declaration of Helsinki. All human volunteers involved in this study provided informed consent. Human platelet suspensions were prepared as described previously^34^. Human blood samples were obtained from adult volunteers who refrained from the use of drugs or other substances that could interfere with the experiment for at least 14 days before collection; the collected blood samples were mixed with an acid-citrate-dextrose solution. After centrifugation, the platelet-rich plasma was supplemented with 0.5 μM PGE_1_ and 6.4 IU/ml heparin. Tyrode’s solution containing 3.5 mg/mL BSA was used to prepare the final suspension of washed human platelets. The final Ca^2+^ concentration in Tyrode’s solution was 1 mM. The platelet aggregation test was performed using a lumiaggregometer (Payton Associates, Scarborough, ON, Canada) as described previously^34^. Various concentrations of TQ-6 or a solvent control (0.5% DMSO) were preincubated with platelet suspensions (3.6 × 10^8^ cells/ml) for 3 min before the addition of various concentrations of agonists (i.e., collagen). The extent of platelet aggregation was calculated as a percentage of the control (absence of TQ-6) of light-transmission units, after the reaction proceeded for 6 min. For ATP release assay tests, 20 μl of luciferin-luciferase was added 1 min before the addition of the agonist; the amount of ATP released was compared with that released by the control.

### Measurement of intracellular [Ca^2+^]i mobilization by using Fura-2AM fluorescence

The amount of intracellular calcium concentration [Ca^2+^]i was determined using Fura-2AM as described previously^34^. Briefly, citrated whole blood was centrifuged at 120 × g for 10 min; the supernatant was collected and incubated with 5 μM Fura-2AM for 1 h. Human platelets were prepared as described in the previous section. The Fura-2AM-loaded platelets were washed and preincubated with TQ-6 in the presence of 1 mM CaCl_2_ and then stimulated with collagen. The Fura-2 fluorescence was measured using a spectrofluorometer (Hitachi FL Spectrophotometer F-4500, Tokyo, Japan) at excitation wavelengths of 340 and 380 nm and an emission wavelength of 510 nm.

### Detection of lactate dehydrogenase (LDH)

Washed platelets (3.6 × 10^8^ cells/ml) were preincubated with 20, 50, and 100 μM TQ-6 or the solvent control (0.5% DMSO) for 20 min at 37 °C. An aliquot of the supernatant (10 µl) was deposited on a Fuji Dri-Chem slide LDH-PIII (Fuji, Tokyo, Japan), and the absorbance wavelength was read at 540 nm by using an ultraviolet-visible spectrophotometer (UV-160; Shimadzu, Japan). A maximal value of LDH was noted in sonicated platelets.

### Flow cytometric analysis

Platelet P-selectin, GP VI, and integrin α_IIb_β_3_ expression were performed by flow cytometric analysis. Fluorescence-conjugated triflavin, a specific integrin α_IIb_β_3_ antagonist, was prepared as described previously^15^. The final concentration of FITC-conjugated triflavin was adjusted to 1 mg/ml. Washed platelets were prepared as described in the previous section. The aliquots of platelet suspensions (3.6 × 10^8^ cells/ml) were preincubated TQ-6 (0.3 and 0.5 µM) with FITC-P-selectin (2 µg/ml), FITC-JAQ1 (1 µg/ml), FITC-PAC-1 (2 µg/ml), and FITC-triflavin (2 µg/ml) for 3 min, followed by the addition of collagen (1 µg/ml) or not. The suspensions were then assayed for fluorescein-labeled platelets using a flow cytometer (FACScan Syst., Becton Dickinson, San Jose, CA). Data were collected from 50,000 platelets per experimental group, and the platelets were identified on the basis of their characteristic forward and orthogonal light-scattering profiles. All experiments were repeated at least four times to ensure reproducibility.

### Immunoblotting

Washed platelets (1.2 × 10^9^ cells/ml) were preincubated with various concentrations of TQ-6 or the solvent control (0.5% DMSO) for 3 min, followed by the addition of either collagen to trigger platelet activation or added to immobilized fibrinogen dishes (6-cm), which were pre-coated with fibrinogen (100 μg/ml) overnight at room temperature and then blocked with 1% BSA. The reaction was then stopped, and the platelets were immediately resuspended in 200 μl of lysis buffer. Samples containing 80 μg of protein were separated through 12% SDS gel electrophoresis, and the proteins were electrotransferred to PVDF membranes by using a Bio-Rad semidry transfer unit (Bio-Rad, Hercules, CA, USA). The blots were then blocked with Tris-buffered saline in Tween 20 (TBST; 10 mM Tris-base, 100 mM NaCl, and 0.01% Tween 20) containing 5% BSA for 1 h and probed with various primary antibodies. The membranes were incubated with HRP-linked anti-mouse IgG or anti-rabbit IgG (diluted 1:3000 in TBST) for 1 h. An enhanced chemiluminescence system was used to detect immunoreactive bands, and their optical density was quantified using Bio-profil Biolight (version V2000.01; Vilber Lourmat, Marne-la-Vallée, France).

### Measurement of cyclic nucleotide formation

Platelet suspensions (3.6 × 10^8^ cells/ml) were incubated with 0.1 μM PGE_1_ or 10 μM NTG and 0.3 or 0.5 μM TQ-6 in the presence of 100 μM 3-isobutyl-1-methylxanthine for 6 min. Incubation was stopped, and the solution was immediately boiled for 5 min. The supernatants (50 μl) were employed in determining the contents of cyclic AMP and cyclic GMP by using EIA kits.

### Measurement of hydroxyl radicals through electron spin resonance spectrometry

Electron spin resonance (ESR) spectrometry was performed on a Bruker EMX ESR spectrometer (Bruker, Billerica, MA, USA) as described previously^35^. Platelet suspensions (3.6 × 10^8^ cells/ml) were preincubated with 0.3 or 0.5 μM TQ-6 or 0.5% DMSO for 3 min before the addition of 1 μg/ml collagen. After the suspensions were incubated for 5 min, 100 μM DMPO was added before the execution of ESR spectrometry. ESR spectra were recorded at room temperature using quartz flat cell designed for water solution. The dead time between platelet suspension and ESR analysis was exactly 30 s after the last addition. The spectrometer was operated at a power of 20 mW, frequency of 9.78 GHz, scan range of 100 G, and receiver gain of 5 × 10^4^. Modulation amplitudes, 1 G; time constant, 164 ms; and scanning for 42 s with 3 scans accumulated. The rate of OH**·** scavenging activity of TQ-6 was calculated as follows: inhibition rate = 1 – [signal height (TQ-6)/signal height (solvent control)]^35^.

### Platelet function analysis for whole blood

A Dade Behring PFA-100 system (Dade Behring, Marburg, Germany) was used to analyze platelet function^36^. Cartridges containing CEPI-coated membranes were preincubated with various concentrations of TQ-6 or the solvent control (0.5% DMSO) for 2 min. Whole blood aliquots (0.8 ml/cartridge) were applied to the cartridges before the contents were exposed to high-shear-flow conditions (5000–6000/s). Closure time (CT) was defined as the time required for a platelet plug to occlude the aperture in the collagen membrane^36^.

### Measurement of sodium fluorescein-induced platelet thrombus formation in mice mesenteric microvessels

Male ICR mice (6 weeks) were anesthetized using a mixture containing 75% air and 3% isoflurane maintained in 25% oxygen; their external jugular veins were cannulated with a PE-10 tube for administering the dye and drugs intravenously^37^. Venules (30–40 µm) were irradiated at wavelengths of <520 nm to produce a microthrombus. Two doses of TQ-6 (0.2 and 0.4 mg/kg) were administered 1 min following sodium fluorescein (15 µg/kg) administration, and the time required for the thrombus to occlude the microvessel (occlusion time) was recorded. In this experiment, the method applied on the thrombogenic animal model conformed to the Guide for the Care and Use of Laboratory Animals (8th edition, 2011) and was approved by Affidavit of Approval of Animal Use Protocol-Taipei Medical University (LAC-2016–0395).

### Measurement of bleeding time in mouse tail vein

The bleeding time was measured through transection of the tail of the mice. In brief, after 30 min of administering either 0.4 mg/kg TQ-6 or 150 mg/kg aspirin intraperitoneally, we sharply cut the tail of the mice at 3 mm from the tip. The tails were immediately placed into a tube filled with saline at 37 °C for measuring bleeding time, which was recorded until the bleeding completely stopped.

### Statistical analysis

The experimental results are expressed as means ± standard errors of the means and are accompanied by the number of observations (*n*). Values of *n* refer to the number of experiments, and each experiment was conducted using different blood donors. The unpaired Student’s *t* test was used to determine significant differences in the occlusion times and bleeding time of mice. The differences between the groups in other experiments were assessed using ANOVA. When the ANOVA indicated significant differences among the group means, the groups were compared using the Student–Newman–Keuls method. A p value of <0.05 indicated statistical significance. Statistical analyses were performed using SAS (version 9.2; SAS Inc., Cary, NC, USA).
