# Author Correction: A novel ruthenium (II)-derived organometallic compound, TQ-6, potently inhibits platelet aggregation: *Ex vivo* and *in vivo* studies

**DOI:** 10.1038/s41598-020-58043-1

**Published:** 2020-01-29

**Authors:** Chih-Hsuan Hsia, Marappan Velusamy, Joen-Rong Sheu, Themmila Khamrang, Thanasekaran Jayakumar, Wan-Jung Lu, Kuan-Hung Lin, Chao-Chien Chang

**Affiliations:** 1 Graduate Institute of Medical Sciences, College of Medicine, Taipei Medical University, Taipei, 110 Taiwan; 2 Department of Chemistry, North Eastern Hill University, Shillong, 793022 India; 3 Department of Medical Research, Taipei Medical University Hospital, Taipei, 110 Taiwan; 4 Central Laboratory, Shin-Kong Wu Ho-Su Memorial Hospital, Taipei, 111 Taiwan; 5 Department of Pharmacology, School of Medicine, College of Medicine, Taipei Medical University, Taipei, 110 Taiwan; 6 Department of Cardiology, Cathay General Hospital, Taipei, 106 Taiwan

Correction to: *Scientific Reports* 10.1038/s41598-017-09695-z, published online 25 August 2017

The original version of this Article omitted an additional affiliation for Wan-Jung Lu. The correct affiliations for Wan-Jung Lu are listed below:

Graduate Institute of Medical Sciences, College of Medicine, Taipei Medical University, Taipei, 110, Taiwan

Department of Medical Research, Taipei Medical University Hospital, Taipei, 110, Taiwan

Department of Pharmacology, School of Medicine, College of Medicine, Taipei Medical University, Taipei, 110, Taiwan

This error has now been corrected in the HTML and PDF versions of the article.

